# Lectures on the Treatment of Fibroid Tumors of the Uterus

**Published:** 1898-07

**Authors:** 


Lectures on the Treatment of Fibroid Tumors of the Uterus. By Franklin H. Martin, M. D., Professor of Gynecology at the Post-Graduate Medical School, Chicago, etc. Chicago. W. T. Keener & Co. 1897. Pages, 165. Price, cloth, $1.

A course of lectures upon the etiology, pathology and treatment of uterine fibroids. Proper attention is given to all the details, the work is well written and fully covers the subject.

J.

---

The *Review of Reviews* continues strong on war topics. In the July number the editor reviews the whole campaign up to the landing of our troops for the advance on Santiago, showing the precise part which Lieutenant Hobson's exploit had in the general scheme; Dr. William Hayes Ward treats of Hobson's career as that of the typical young American student; Mr. Edwin Emerson, Jr., the brilliant young newspaper correspondent, gives notes of his adventurous journeyings in Porto Rico last month; and Dr. Max West, the statistician and economist, summarizes "Our New War Taxes" in an interesting article. "International Cartoon Comments on Our War with Spain" and the "Record of Current Events" also cover the situation up to date.

---

**At the Denver Meeting** of the American Medical Association the only complaints made by the delegates was on the score of the hotels and hackmen; both furnished poor service and charged exorbitant rates. A few of us were fortunate enough to escape a crowded hotel where both the service and the fare are poor, because, as the manager explains, of the "rush." We had the good fortune to put up at the "Home," where everything was pleasant and homelike. It is not a hotel, but has all the comforts and conveniences of one, with many advantages added. In the first place you get a perfectly clean room, one that has been fumigated and revarnished since the last guest vacated. You get better service and decidedly more prompt attention than at a hotel, and the table fare is the best and most wholesome, and what is more is absolutely clean. You can eat with a relish, feeling that the food is so prepared as to be the most digestible. We would advise any of our friends who expect to spend a week or more in Denver to write to the superintendent of the Home, Rev. Frederick W. Oakes, 2903 Fairview Ave., Denver, Colorado.

